# Targeting the Fatty Acid Binding Protein 5–Specificity Protein 1 Axis Restores Enzalutamide Sensitivity by Suppressing Androgen Receptor/Androgen Receptor Splice Variant 7 Signaling: Implications for Prostate Cancer Therapy

**DOI:** 10.14740/wjon2799

**Published:** 2026-07-30

**Authors:** Saud A. Abdulsamad, Abdulghani A. Naeem, Ahmaed Baashar, Ahmed A. Aldarmahi, Faisal F. Alamri, Nada K. Abuarab, Zaki Alsahafi, Ghaith Fallata, Anas Bokhari, Abdulmajeed H. Alharbi, Youqiang Ke, Gang He

**Affiliations:** a Department of Basic Sciences, College of Science and Health Professions, King Saud bin Abdulaziz University for Health Sciences, Jeddah, Saudi Arabia; b King Abdullah International Medical Research Center, Jeddah, Saudi Arabia; c Department of Molecular and Clinical Cancer Medicine, Liverpool University, Liverpool L69 3PX, UK; d Key Laboratory of Medicinal and Edible Plants Resources Development of Sichuan Education Department, Sichuan Industrial Institute of Antibiotics, School of Pharmacy, Chengdu University, Chengdu 610106, China

**Keywords:** Prostate cancer, Castration-resistant prostate cancer, FABP5–Sp1 axis, Androgen receptor, AR-V7, Enzalutamide resistance, Enzalutamide sensitivity, Sp1 inhibition, PPARγ signaling, VEGFA

## Abstract

**Background:**

Castration-resistant prostate cancer (CRPC) remains a major clinical challenge driven by persistent androgen receptor (AR) signaling and constitutively active splice variants such as androgen receptor splice variant 7 (AR-V7), which confer resistance to therapies including enzalutamide. Although metabolic reprogramming contributes to disease progression, the integration of metabolic and transcriptional regulators sustaining therapeutic resistance remains incompletely understood.

**Methods:**

We integrated clinical transcriptomic analysis of The Cancer Genome Atlas Prostate Adenocarcinoma (TCGA-PRAD) cohort with mechanistic and functional validation in 22RV1 CRPC cells to investigate the role of the fatty acid binding protein 5–specificity protein 1 (FABP5–Sp1) regulatory axis.

**Results:**

Transcriptomic analysis revealed that FABP5 is significantly upregulated in prostate tumors compared with normal tissue and increases with higher Gleason score. In contrast, AR and Sp1 exhibited heterogeneous expression patterns. Mechanistically, genetic ablation of FABP5 markedly reduced AR-V7 expression and restored sensitivity to enzalutamide, leading to suppression of AR signaling. Conversely, FABP5 overexpression increased Sp1 protein levels. Pharmacological inhibition of Sp1 using mithramycin A resulted in coordinated downregulation of FABP5, AR, and AR-V7, along with suppression of peroxisome proliferator-activated receptor gamma (PPARγ) signaling and downstream vascular endothelial growth factor A (VEGFA) expression. Functionally, Sp1 inhibition significantly reduced anchorage-independent growth and invasion.

**Conclusion:**

These findings define a FABP5–Sp1–AR/AR-V7 transcriptional–metabolic axis driving enzalutamide resistance in CRPC. Targeting FABP5 restores therapeutic sensitivity and represents a promising biomarker and therapeutic strategy in advanced prostate cancer.

## Introduction

Prostate cancer (PC) ranks as the second most common diagnosed cancer and the fifth leading cause of cancer-related mortality among men globally, with approximately 1.46 million new cases and over 396,000 deaths reported in 2022. Records indicate that, driven primarily by population growth and aging, the global burden of PC will rise substantially by 2040, reaching approximately 2.4 million new cases and 712,000 deaths [1, 2]. Within Saudi Arabia, PC is the most frequently reported malignancy among males aged 60 and above and ranks as the third most common cancer in men overall, exhibiting steadily increasing incidence rates attributed to increased life expectancy and enhanced screening programs [3]. PC manifests as a heterogeneous disease, initially dependent on androgen signaling for proliferation and survival, thus classified as an androgen-dependent malignancy [4, 5]. During this initial phase, prostate tumors exhibit a high degree of responsiveness to androgen deprivation therapy (ADT), which functions by suppressing circulating testosterone levels and impairing androgen receptor (AR) activity [6–8]. Nevertheless, despite the initial clinical benefits, the majority of patients ultimately progress to an androgen-independent state, clinically defined as castration-resistant prostate cancer (CRPC) [9, 10]. Despite diminished androgen levels, CRPC persists by reactivating AR signaling through mechanisms such as AR gene amplification, point mutations, increased intratumoral androgen synthesis, and the expression of constitutively active AR splice variants, including androgen receptor splice variant 7 (AR-V7) [11–14]. In parallel with experimental studies, emerging transcriptomic analyses of patient-derived PC cohorts have highlighted dysregulation of lipid metabolism and transcriptional control pathways in advanced disease [15–17]. Notably, altered expression of fatty acid binding protein 5 (FABP5) and specificity protein 1 (Sp1) has been reported in aggressive prostate tumors and metastatic settings, suggesting a potential clinical role for this axis in sustaining AR signaling [18–20]. However, while AR-V7 has been clinically linked to resistance to AR-targeted therapy [21–23], enzalutamide is a second-generation AR inhibitor that blocks androgen binding, prevents AR nuclear translocation, and inhibits AR-mediated transcriptional activity [24, 25], and FABP5 expression has been associated with aggressive tumor features including higher Gleason grade [18, 26, 27]. The integration of FABP5–Sp1 regulation with AR-V7 signaling within a unified clinical framework remains insufficiently defined [21]. Despite these clinical observations, the mechanistic basis linking FABP5 and Sp1 to persistent AR and AR-V7 signaling has not been fully elucidated. In particular, it remains unclear whether FABP5 and Sp1 function as a coordinated regulatory axis capable of sustaining AR signaling under therapeutic pressure, including enzalutamide treatment [28, 29]. Addressing this gap requires integration of clinical transcriptomic evidence with mechanistic validation in relevant CRPC models [15, 16]. The 22RV1 cell line is a frequently employed model in CRPC studies. Derived from the CWR22R xenograft following castration relapse, these cells, unlike LNCaP or VCaP cells, exhibit endogenous expression of both full-length AR and the AR-V7 variant [30–32]. This characteristic renders them an appropriate in vitro model for elucidating the molecular mechanisms underpinning AR signaling and drug resistance in CRPC [33]. Their co-expression profile is particularly valuable for assessing the efficacy of next-generation AR-targeted therapies and for investigating the mechanisms of resistance mediated by splice variants [34, 35]. Recent investigations have identified Sp1, a zinc-finger transcription factor, as a pivotal regulator influencing the expression of both FABP5 and AR. Sp1, which binds to GC-rich motifs located in the promoter regions of numerous oncogenes, is recognized for its role in governing genes associated with proliferation, angiogenesis, and hormonal responses [20, 36–38]. Notably, vascular endothelial growth factor A (VEGFA), a key mediator of tumor angiogenesis, has been reported to be regulated through lipid signaling pathways, including peroxisome proliferator-activated receptor gamma (PPARγ)-dependent mechanisms in PC. Elevated Sp1 expression in PCa correlates with disease progression [37]. Sp1 was selected for investigation in the present study because previous studies have demonstrated its ability to regulate both AR and FABP5 expression, positioning it as a potential molecular link between metabolic signaling and AR-driven therapeutic resistance [39]. Notably, Sp1 has been demonstrated to regulate AR gene expression and potentially modulates alternative splicing, leading to the production of variants such as AR-V7 [39, 40]. The presence of a CpG-rich island in the FABP5 promoter further supports the possibility of Sp1-mediated transcriptional regulation. Collectively, these findings demonstrate that the presence of a FABP5–Sp1–AR regulatory mechanism may play a significant role in maintaining AR signaling and promoting resistance to enzalutamide in CRPC [18, 36, 41, 42]. Further, the functional significance of this mechanism in the context of AR-V7-mediated resistance warrants further systematic investigation [11, 43]. Based on these considerations, this study aimed to integrate clinical transcriptomic evidence with mechanistic validation to define the role of the FABP5–Sp1 axis in regulating AR and AR-V7 signaling in CRPC. Specifically, we assessed the clinical relevance of FABP5 and Sp1 expression in patient-derived datasets and subsequently employed CRPC cell models to mechanistically examine how disruption of this axis influences AR signaling and responsiveness to enzalutamide. However, whether this axis functionally regulates downstream oncogenic phenotypes such as invasion, anchorage-independent growth, and angiogenic signaling, including VEGFA, remains unclear.

## Materials and Methods

### Cell culture

The PC cell line 22RV1 (ATCC, USA) and FABP5 knockout (FABP5-KO) derivatives were cultured in RPMI 1640 (Gibco; Thermo Fisher Scientific, Inc.) medium, which was supplemented with 2 mM of L-glutamine, 10% (v/v) fetal bovine serum (FBS), and 100 U/mL of penicillin/streptomycin (Sigma-Aldrich; Merck KGaA). The cells were kept in a controlled environment—a humidified incubator set at 37 °C, with a mixture of 5% CO_2_ and 95% air. Culture maintenance involved refreshing the media every 3 days and sustaining the cells as monolayers.

### Treatment preparation and experimental exposure

Recombinant FABP5 protein preparation was performed as previously described [27]. Enzalutamide and mithramycin A (MedChemExpress) were dissolved in dimethyl sulfoxide (DMSO) to generate 10 mM stock solutions, aliquoted, and stored at −20 °C to avoid repeated freeze–thaw cycles. For experimental treatments, cells were exposed to vehicle control, 10 µM enzalutamide, 5 µM recombinant FABP5 protein, or 1 µM mithramycin A. Cells were exposed to the indicated treatments for 24 h prior to protein extraction and downstream analyses. A single concentration of mithramycin A (1 µM) was selected based on previous studies demonstrating effective inhibition of Sp1 transcriptional activity within this range, while maintaining acceptable cell viability. This concentration enabled focused evaluation of downstream molecular and functional effects associated with Sp1 suppression. These experimental conditions were selected to interrogate the regulatory relationship between FABP5, Sp1, and AR signaling under AR-targeted therapeutic pressure. Following treatment, total cellular protein was extracted for downstream analyses.

### Soft agar colony formation assay

To evaluate anchorage-independent growth, a soft agar colony formation assay was performed using 22RV1 cells. Six-well plates were pre-coated with 1% low-melting agarose in complete RPMI 1640 medium and allowed to solidify.

Cells (5 × 10^4^ per well) were suspended in 0.5% agarose containing complete medium and treated with either vehicle control (DMSO) or 1 µM mithramycin A, then layered onto the base agar. Plates were incubated at 37 °C in a humidified atmosphere with 5% CO_2_ for 21 days, with fresh medium containing the respective treatments added every 3–4 days. At the end of the incubation period, colonies were stained with crystal violet and counted under a light microscope. Experiments were performed in at least three independent biological replicates.

### Cell invasion assay

Cell invasion was assessed using Matrigel-coated Transwell chambers (8 µm pore size). The lower chamber was filled with complete RPMI 1640 medium containing 10% FBS as a chemoattractant. The 22RV1 cells (2.5 × 10^4^) were seeded in the upper chamber in serum-free medium and treated with either DMSO (control) or 1 µM mithramycin A. After 24 h of incubation at 37 °C, non-invading cells were removed, and invading cells were fixed, stained with crystal violet, and counted under a microscope. Invaded cells were quantified from randomly selected fields, and all experiments were performed in triplicate.

### Protein quantification and sample preparation

Total protein concentration was determined using the Bradford assay. Briefly, protein samples and bovine serum albumin standards were incubated with Coomassie Brilliant Blue G-250 reagent, and absorbance was measured at 595 nm. For electrophoresis, protein samples were mixed with Laemmli buffer containing β-mercaptoethanol, heated at 95 °C for 10 min, cooled on ice, and loaded onto sodium dodecyl sulphate-polyacrylamide gels. Electrophoresis was performed at 150 V for 40–60 min.

### Western blot and antibody detection

Proteins were transferred onto polyvinylidene fluoride membranes (Immobilon-P, Millipore, USA) using a Bio-Rad Mini-Protein transfer system at 100 V for 1 h in cold transfer buffer. Membranes were activated in methanol, equilibrated in transfer buffer, and blocked with 5% non-fat milk in Tris-buffered saline Tween (TBST) for 1 h at room temperature. Primary antibodies against AR, AR-V7, Sp1, FABP5, PPARγ, phosphorylated PPARγ (p-PPARγ), VEGFA, and β-actin were incubated overnight at 4 °C. After washing, membranes were incubated with horseradish peroxidase (HRP)-conjugated secondary antibodies for 1–2 h at room temperature. Protein bands were visualized using enhanced chemiluminescence (Immobilon ECL Ultra, Millipore) and detected with a ChemiDoc MP Imaging System (Bio-Rad). Band intensities were quantified using ImageJ software and normalized to β-actin to correct for loading variability. Details of the primary and secondary antibodies used in this study, including suppliers and dilution factors, are summarized in Table 1.

**Table 1:** Details of the Primary and Secondary Antibodies Used in This Study

Target protein	Primary antibody (dilution, supplier)	Secondary antibody (dilution, supplier)
FABP5	Rabbit monoclonal (1:500, Hycult Biotech)	Swine anti-rabbit polyclonal HRP (1:10,000, DAKO)
AR	Mouse monoclonal (1:400, Santa Cruz Biotechnology)	Rabbit anti-mouse polyclonal HRP (1:10,000, DAKO)
AR-V7	Rabbit polyclonal (1:1000, Cell Signaling Technology)	Swine anti-rabbit polyclonal HRP (1:10,000, DAKO)
Sp1	Rabbit polyclonal (1:1000, Cell Signaling Technology)	Swine anti-rabbit polyclonal HRP (1:10,000, DAKO)
PPARγ	Mouse monoclonal (1:500, Santa Cruz Biotechnology)	Rabbit anti-mouse polyclonal HRP (1:10,000, DAKO)
p-PPARγ (Ser112)	Rabbit polyclonal (1:250, ThermoFisher Scientific)	Swine anti-rabbit polyclonal HRP (1:10,000, DAKO)
VEGFA	Rabbit polyclonal (1: 1,000) (Abcam)	Swine anti-rabbit polyclonal HRP (1:10,000, DAKO)
β-actin	Mouse monoclonal (1:20,000, Abcam)	Rabbit anti-mouse polyclonal HRP (1:20,000, Abcam)

AR: androgen receptor; AR-V7: androgen receptor splice variant 7; FABP5: fatty acid binding protein 5; HRP: horseradish peroxidase; PPARγ: peroxisome proliferator-activated receptor gamma; p-PPARγ: phosphorylated PPARγ; Sp1: specificity protein 1; VEGFA: vascular endothelial growth factor A.

### Clinical transcriptomic data analysis

Publicly available PC transcriptomic datasets were analyzed to evaluate the clinical relevance of FABP5, Sp1, and AR signaling components. Gene expression data were obtained from The Cancer Genome Atlas Prostate Adenocarcinoma cohort (TCGA-PRAD) and selected publicly available Gene Expression Omnibus (GEO) datasets. Expression levels were compared between normal prostate tissues and primary prostate adenocarcinoma samples, as well as across available clinic pathological parameters, including Gleason score categories.

TCGA-PRAD RNA-sequencing data accessed through GEPIA2 and cBioPortal are pre-processed and normalized by the original TCGA analysis pipelines and were analyzed as provided. Correlation analyses were performed using Pearson correlation coefficients, as appropriate. Data visualization and statistical analyses were conducted using the cBioPortal and GEPIA2 platforms.

### Statistical analysis

Statistical analyses were performed using GraphPad Prism (version 9) and Microsoft Excel. Western blot band intensities were quantified using ImageJ software. Experimental data are presented as mean ± standard deviation (SD) from at least three independent biological replicates (n = 3). Comparisons between groups were conducted using an unpaired two-tailed Student’s t-test. For clinical transcriptomic analyses, correlation significance was assessed using Pearson correlation coefficients. A P-value < 0.05 was considered statistically significant.

### Ethical compliance with human/animal study

This study did not involve human participants, patient samples, or animal experiments. Publicly available transcriptomic datasets were analyzed in accordance with relevant database usage guidelines.

## Results

### Differential expression of FABP5, Sp1, and AR in PRAD tumors versus normal prostate tissue

**Figure 1:**
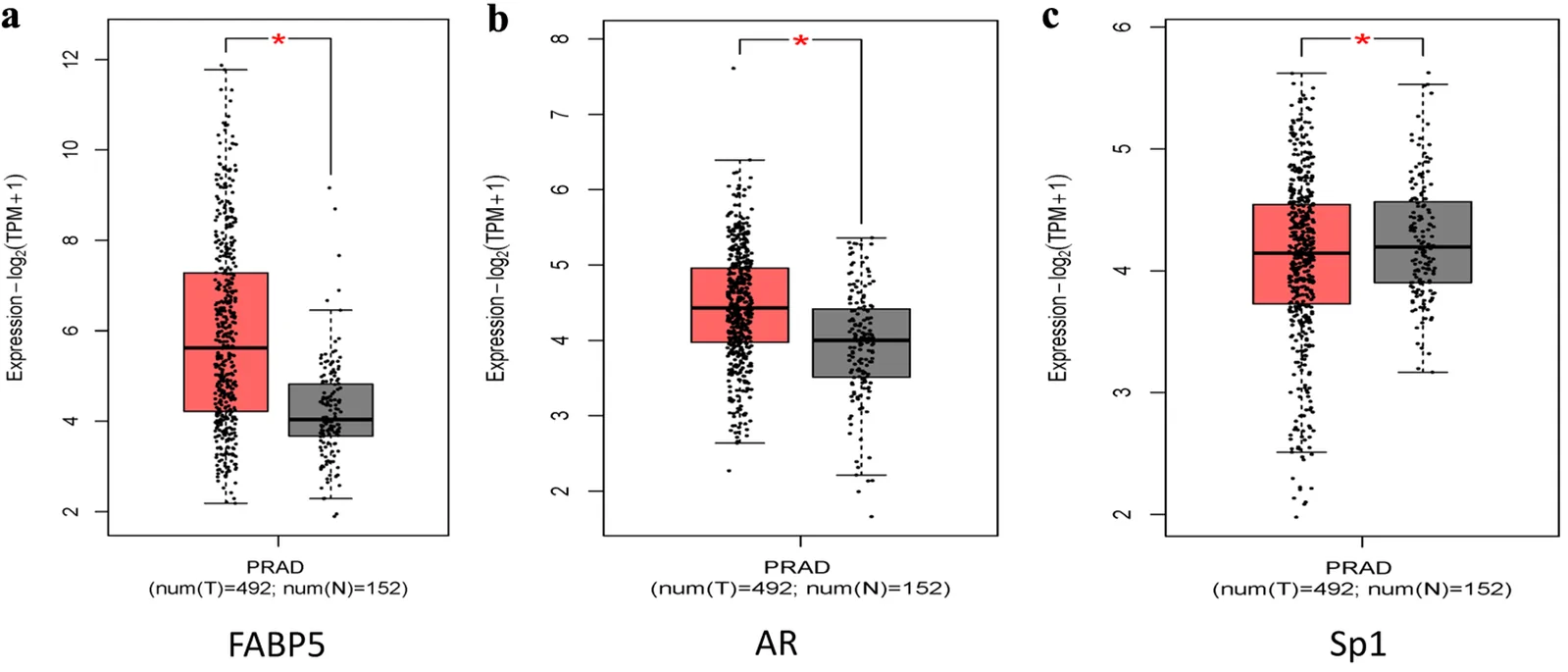
Differential expression of FABP5, AR, and Sp1 between normal prostate tissue and primary PRAD. (a) FABP5 expression is significantly elevated in primary tumor samples compared with normal prostate tissue. (b) AR expression shows a modest but significant reduction in tumor samples relative to normal tissue. (c) Sp1 expression is moderately decreased in tumor samples compared with normal prostate tissue. Gene expression levels are presented as log_2_(TPM + 1). Data were obtained from TCGA-PRAD datasets and visualized using GEPIA2. Statistical significance was determined using Student’s t-test. *P < 0.05, **P < 0.01, ***P < 0.001. AR: androgen receptor; FABP5: fatty acid binding protein 5; Sp1: specificity protein 1; PRAD: prostate adenocarcinoma.

Analysis of TCGA-PRAD transcriptomic data revealed differential expression patterns of FABP5, AR, and Sp1 between normal prostate tissues and primary prostate adenocarcinoma samples (Fig. 1a–c). FABP5 expression was markedly elevated in tumor samples compared with normal prostate tissue (Fig. 1a; P < 0.001), indicating tumor-associated upregulation. In contrast, AR expression showed a modest reduction in primary tumor samples relative to normal tissue (Fig. 1b; P < 0.01), while Sp1 exhibited a moderate decrease in expression in tumors compared with normal prostate samples (Fig. 1c; P < 0.01). These findings highlight gene-specific expression alterations associated with prostate tumorigenesis and suggest differential regulatory roles for FABP5, AR, and Sp1 in PC.

### Association of FABP5, Sp1, and AR expression with PC grade (Gleason score)

**Figure 2:**
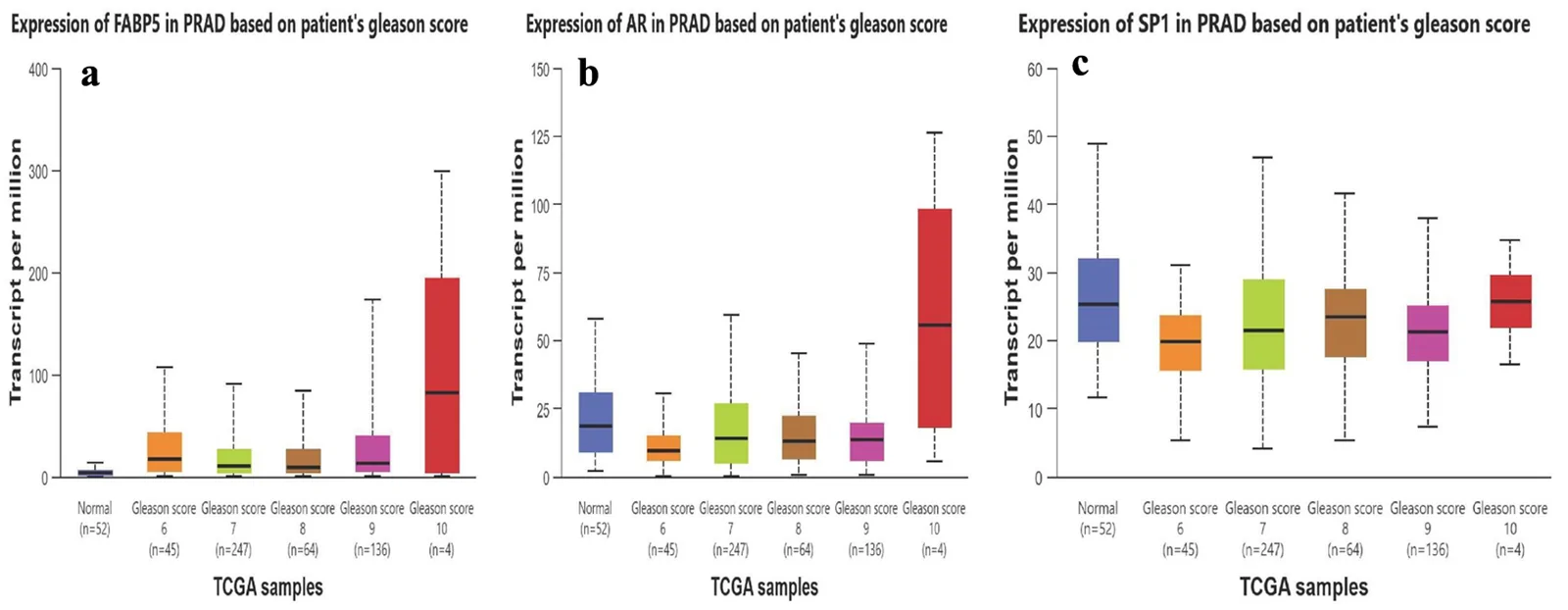
Expression patterns of FABP5, AR, and Sp1 across prostate cancer Gleason score groups in the TCGA-PRAD. (a) FABP5 expression progressively increases with higher Gleason score categories, with the highest levels observed in high-grade tumors. (b) AR expression demonstrates an overall increase in higher Gleason score groups, consistent with its association with advanced disease. (c) Sp1 expression shows moderate variability across Gleason score categories without a consistent monotonic trend. Gene expression levels are presented as transcripts per million (TPM). Data and associated statistical analyses were obtained from the TCGA-PRAD dataset through the UALCAN platform. AR: androgen receptor; FABP5: fatty acid binding protein 5; Sp1: specificity protein 1.

Stratification of TCGA-PRAD samples according to Gleason score demonstrated distinct expression patterns for FABP5, AR, and Sp1 (Fig. 2a–c). FABP5 expression exhibited a progressive increase with advancing tumor grade, with significant differences observed in Gleason score 6 (P < 0.01), Gleason score 7 (P < 0.0001), Gleason score 8 (P < 0.01), and Gleason score 9 (P < 0.001) compared with normal tissue (Fig. 2a). AR expression also increased in higher-grade tumors, with a significant difference observed in Gleason score 6 (P < 0.01) (Fig. 2b). In contrast, Sp1 expression displayed moderate variability, with significant changes detected in Gleason score 6 (P < 0.001), Gleason score 7 (P < 0.05), and Gleason score 9 (P < 0.001), but no consistent trend across all grades (Fig. 2c).

### Sp1 inhibition suppresses tumorigenic phenotypes in 22RV1 cells

**Figure 3:**
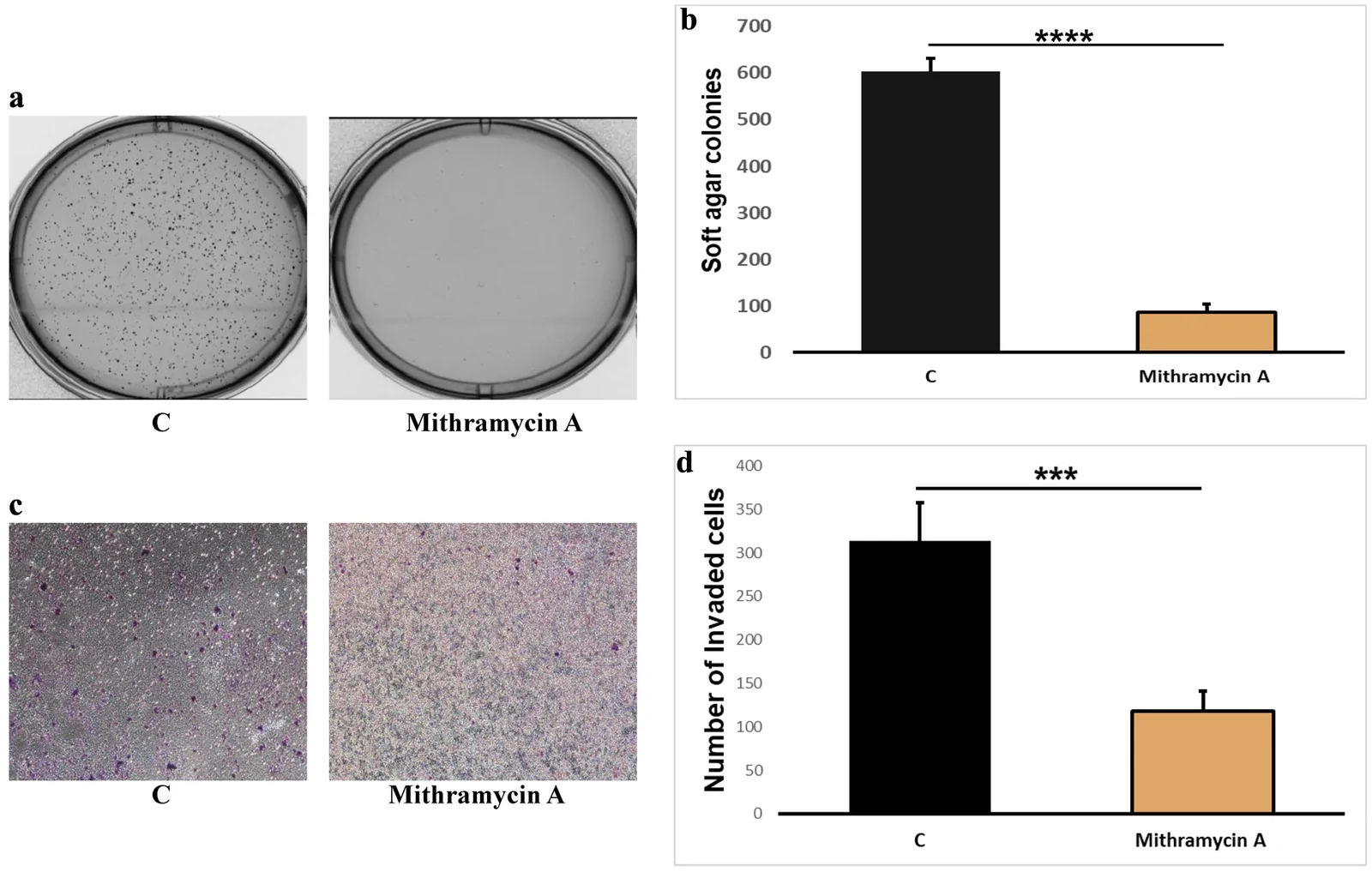
Sp1 inhibition by mithramycin A reduces anchorage-independent growth and invasion in 22RV1 cells. (a) Representative soft agar colony formation images of 22RV1 cells treated with DMSO control (C) or 1 µM mithramycin A. (b) Quantification of soft agar colonies showing a significant reduction after mithramycin A treatment. (c) Representative invasion assay images of 22RV1 cells treated with DMSO control (C) or 1 µM mithramycin A. (d) Quantification of invaded cells showing a significant reduction after mithramycin A treatment. Quantitative data in panels (b) and (d) are presented as mean ± SD from three independent experiments. ****P < 0.0001; ***P < 0.001. DMSO: dimethyl sulfoxide; Sp1: specificity protein 1; SD: standard deviation.

To evaluate the functional impact of Sp1 inhibition on tumorigenic phenotypes, we assessed anchorage-independent growth and invasion following mithramycin A treatment. As shown in Figure 3a, representative soft agar images demonstrate a marked reduction in colony formation upon Sp1 inhibition. Quantitative analysis (Fig. 3b) revealed that the number of colonies significantly decreased from 603.67 ± 28.10 in control cells to 86.33 ± 17.24 following treatment (****P < 0.0001), indicating impaired tumorigenic potential.

In parallel, invasion assays showed a substantial reduction in invasive capacity. Representative images (Fig. 3c) illustrate fewer invading cells following mithramycin A treatment, while quantification (Fig. 3d) confirmed a significant decrease from 314.00 ± 43.31 in control cells to 118.00 ± 23.07 after treatment (***P < 0.001).

### FABP5-KO enhances enzalutamide response in 22RV1 cells by attenuating AR and AR-V7 expression

**Figure 4:**
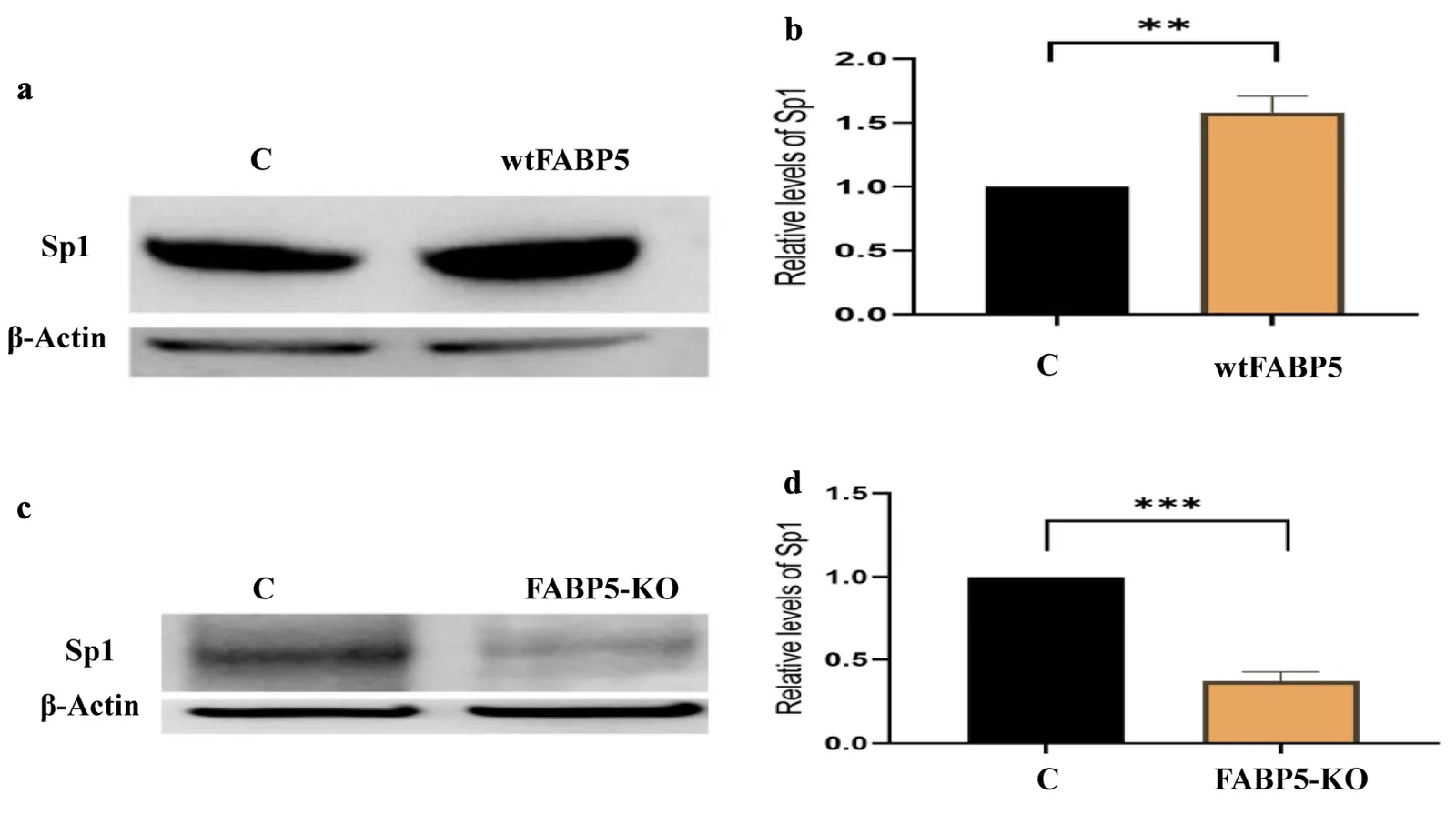
Effect of enzalutamide on AR and AR-V7 expression in both 22RV1 and 22RV1-FABP5 knockout cells. (a) Western blot analysis illustrates the protein levels of full-length AR and AR-V7 in both wild-type and FABP5 knockout 22RV1 cells under both baseline conditions and following enzalutamide treatment. β-Actin was used as a loading control. (b) The quantification of AR and AR-V7 protein expression, normalized to actin levels, is presented as the mean ± SD derived from three independent experimental trials. Statistical significance was determined using unpaired two-tailed Student’s t-test. ****P < 0.0001. AR: androgen receptor; AR-V7: androgen receptor splice variant 7; FABP5: fatty acid binding protein 5.

To investigate the role of FABP5 in modulating AR signaling under therapeutic exposure, we examined AR and AR-V7 expression in parental 22RV1 and 22RV1-FABP5-KO cells treated with enzalutamide (Fig. 4a, b). When expression levels in parental 22RV1 cells were normalized to 1, AR levels in 22RV1-FABP5-KO cells showed no significant change, whereas AR-V7 expression was significantly reduced by 91% to 0.095 ± 0.005 (****P < 0.0001), indicating a strong dependence of AR-V7 on FABP5. As shown in Figure 4a, b, enzalutamide treatment did not significantly alter AR (1.02 ± 0.07) or AR-V7 (0.98 ± 0.05) levels in parental 22RV1 cells. In contrast, in 22RV1-FABP5-KO cells, enzalutamide significantly decreased AR expression by 89% to 0.12 ± 0.01 (****P < 0.0001) and almost completely abolished AR-V7 expression, reducing it by 100% to 0.0001 ± 0.00004 (****P < 0.0001). Collectively, these results demonstrate that FABP5 is a critical regulator of AR and AR-V7 stability and activity, and that FABP5 loss markedly sensitizes PC cells to AR-targeted therapy, providing a mechanistic basis for resistance to enzalutamide.

### FABP5 positively regulates Sp1 protein expression in 22RV1 cells

**Figure 5:**
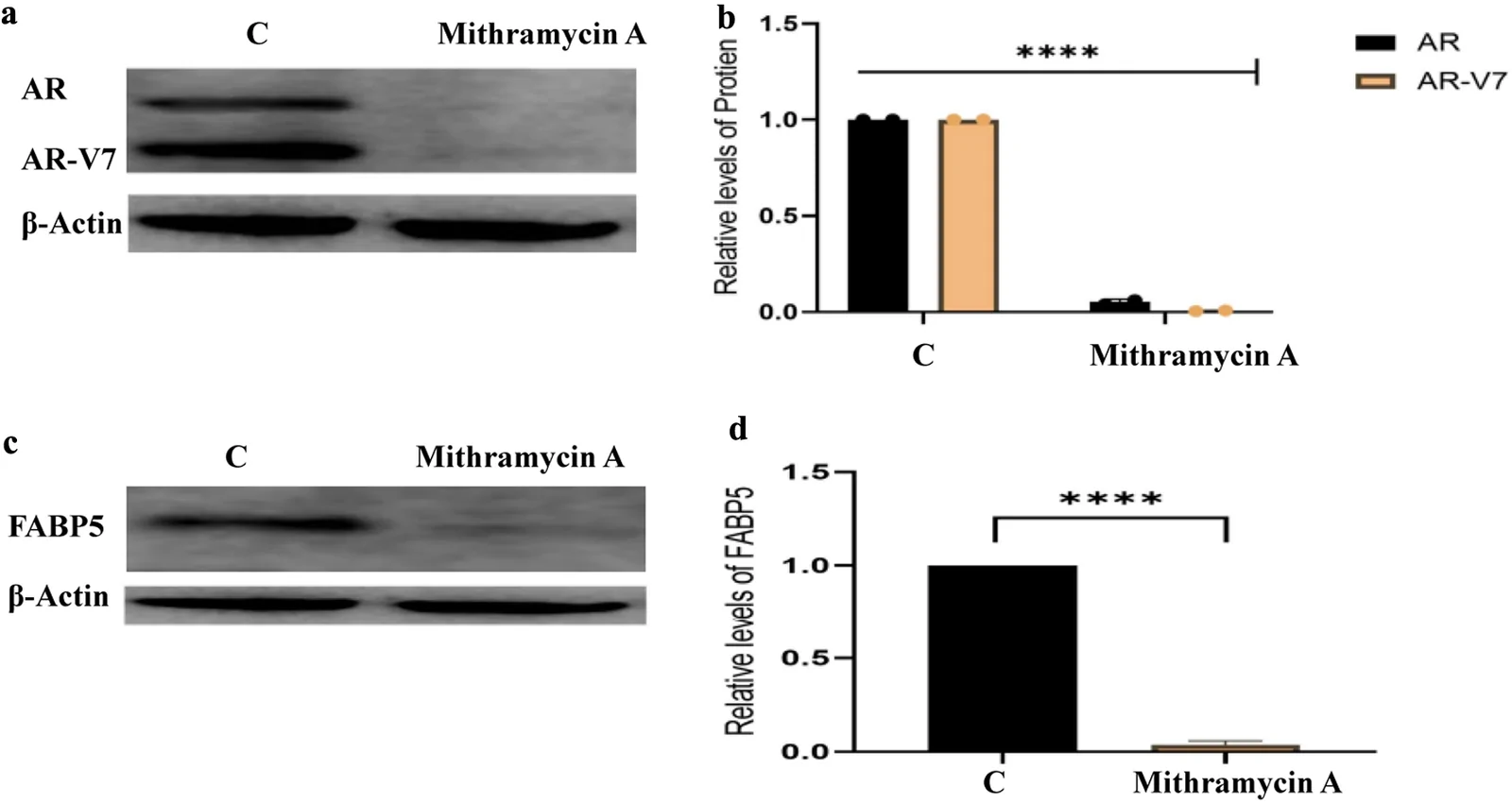
FABP5 positively regulates Sp1 protein expression in 22RV1 cells. (a) Representative Western blot showing increased Sp1 protein levels following FABP5 overexpression in 22RV1 cells compared with control (C). (b) Densitometric quantification of Sp1 expression normalized to β-actin in FABP5-overexpressing cells. (c) Western blot analysis demonstrating reduced Sp1 protein levels in FABP5 knockout (FABP5-KO) 22RV1 cells compared with parental control cells. (d) Quantification of normalized Sp1 protein levels in FABP5-KO cells. Data are presented as mean ± SD from three independent experiments. **P < 0.01; ***P < 0.001. FABP5: fatty acid binding protein 5; Sp1: specificity protein 1; SD: standard deviation.

To further examine FABP5’s functional significance, FABP5 protein was overexpressed in 22RV1 cells. The overexpression was associated with a significant elevation in Sp1 expression (Fig. 5a, b), with Sp1 levels increasing by 58% (1.58 ± 0.09, **P < 0.01) compared with control cells. Sp1 is a transcription factor previously reported to regulate both AR and FABP5 gene expression. In contrast, FABP5-KO led to a substantial reduction in Sp1 protein levels (Fig. 5c, d), with Sp1 expression decreased by 63% (0.37 ± 0.04, ***P < 0.001) relative to parental 22RV1 cells. These findings support a regulatory relationship between FABP5 and Sp1 and suggest bidirectional regulation within this signaling axis.

### Sp1 suppression downregulates AR, AR-V7, and FABP5 expression

**Figure 6:**
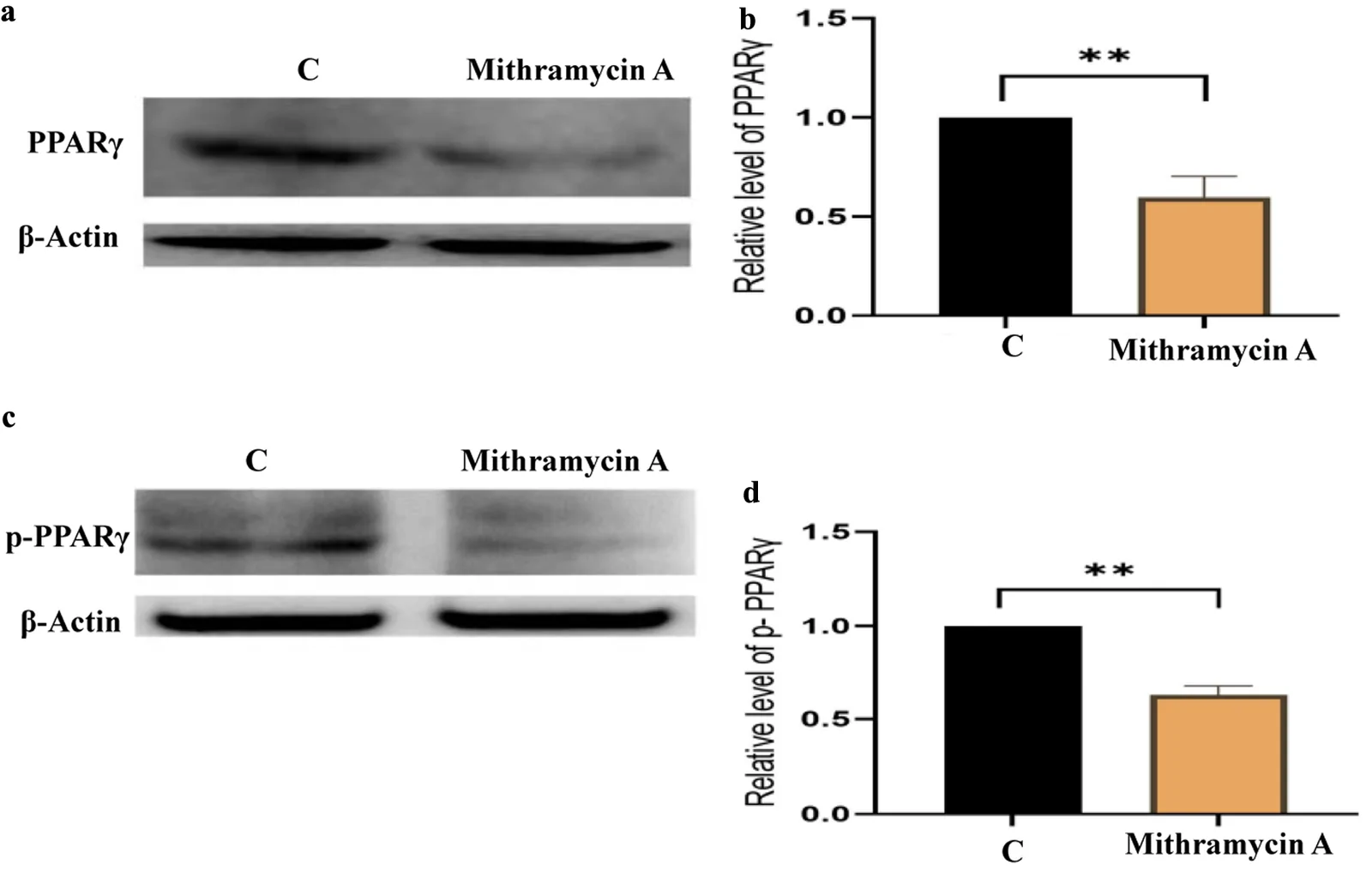
Pharmacological inhibition of Sp1 suppresses AR, AR-V7, and FABP5 expression in 22RV1 cells. (a) Representative Western blot showing reduced AR and AR-V7 protein levels in 22RV1 cells treated with mithramycin A compared with untreated control (C). (b) Densitometric quantification of AR and AR-V7 protein expression normalized to β-actin. (c) Western blot analysis demonstrating marked suppression of FABP5 protein expression following mithramycin A treatment. (d) Quantification of FABP5 protein levels relative to β-actin. Data are presented as mean ± SD from three independent experiments. ****P < 0.0001. AR: androgen receptor; AR-V7: androgen receptor splice variant 7; FABP5: fatty acid binding protein 5; Sp1: specificity protein 1; SD: standard deviation.

To assess the role of Sp1 in regulating AR signaling and FABP5 expression, androgen-responsive 22RV1 cells were treated with 1 µM mithramycin A, with DMSO used as a control. As shown in Fig. 6a, immunoblot analysis, together with densitometric quantification in Figure 6b, demonstrated that mithramycin A treatment significantly reduced AR and AR-V7 expression. When normalized to 1 in control cells, AR and AR-V7 protein levels were decreased by 95% and 99%, reaching 0.052 ± 0.011 and 0.006 ± 0.002, respectively (****P < 0.0001). In addition, mithramycin A treatment almost completely abolished FABP5 expression, as shown by immunoblotting in Figure 6c and corresponding densitometric analysis in Figure 6d. The relative level of FABP5 was reduced by 96% to 0.037 ± 0.016 compared with control cells (****P < 0.0001). Collectively, these data demonstrate that pharmacological inhibition of Sp1 strongly suppresses AR, AR-V7, and FABP5 expression, underscoring the critical role of Sp1 in maintaining this transcriptional axis in 22RV1 PC cells.

### Sp1 inhibition suppresses PPARγ signaling and VEGFA expression in 22RV1 cells

**Figure 7:**
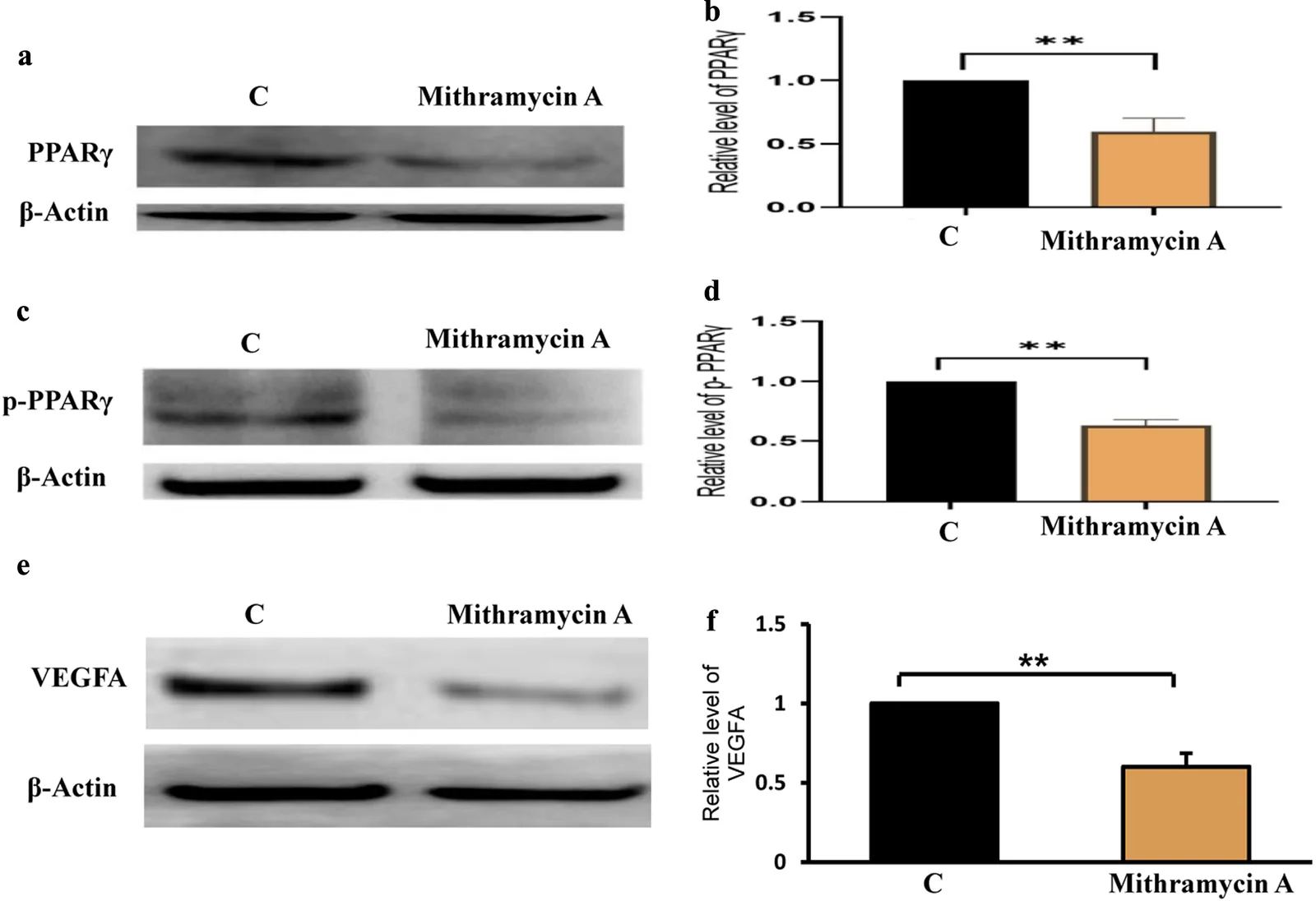
Sp1 inhibition suppresses PPARγ, p-PPARγ signaling, and downstream VEGFA expression in 22RV1 cells. (a) Representative Western blot showing reduced total PPARγ protein levels in 22RV1 cells treated with mithramycin A compared with control (C). (b) Densitometric quantification of total PPARγ expression normalized to β-actin. (c) Western blot analysis demonstrating decreased levels of p-PPARγ following mithramycin A treatment. (d) Quantitative analysis of p-PPARγ protein levels relative to β-actin. Data are presented as mean ± SD from three independent experiments. **P < 0.01. (e) Representative Western blot showing reduced VEGFA protein expression following mithramycin A treatment. (f) Densitometric quantification of VEGFA protein levels normalized to β-actin. Data are presented as mean ± SD from three independent experiments. **P < 0.01. PPARγ: peroxisome proliferator-activated receptor gamma; p-PPARγ: phosphorylated PPARγ; Sp1: specificity protein 1; SD: standard deviation; VEGFA: vascular endothelial growth factor A.

The 22RV1 cells were treated with DMSO (control, C) or 1 µM mithramycin A to assess the effect of Sp1 inhibition on PPARγ signaling. As shown in Figure 7a, Western blot analysis together with densitometry quantification in Figure 7b demonstrated that mithramycin A significantly (P < 0.01) reduced total PPARγ expression by 41%, decreasing its relative level from 1 (control) to 0.59 ± 0.075. Similarly, phosphorylated PPARγ levels were markedly decreased following mithramycin A treatment, as shown in Figure 7c, with densitometric analysis in Figure 7d revealing a significant (P < 0.01) 38% reduction, lowering its relative level from 1 (control) to 0.62 ± 0.035. To further investigate downstream signaling, we examined VEGFA expression following Sp1 inhibition. As shown in Figure 7e, VEGFA protein levels were significantly reduced (P < 0.01) upon mithramycin A treatment, with densitometric quantification in Figure 7f showing an approximate 40% reduction, decreasing from 1 (control) to 0.60 ± 0.086.

Collectively, these findings indicate that pharmacological inhibition of Sp1 suppresses both total and active PPARγ signaling, as well as downstream angiogenic signaling via VEGFA in 22RV1 cells.

## Discussion

PC remains a leading cause of cancer-related morbidity and mortality in men worldwide. Although ADT and next-generation AR inhibitors such as enzalutamide provide significant initial clinical benefit, most patients eventually progress to CRPC, a lethal and treatment-refractory disease state. Reactivation of AR signaling, particularly through constitutively active splice variants such as AR-V7, represents a major mechanism underlying therapeutic resistance. In addition to canonical AR pathway alterations, increasing evidence implicates dysregulated lipid metabolism in sustaining AR activity in advanced disease. Notably, FABP5 has emerged as a key metabolic regulator capable of enhancing nuclear receptor signaling and promoting aggressive PC phenotypes. Understanding how FABP5-dependent metabolic signaling intersects with AR and AR-V7 activation is therefore critical for identifying new therapeutic vulnerabilities in CRPC [18, 41, 44].

In this study, we integrated clinical transcriptomic analyses with mechanistic validation in CRPC cell models to define the role of the FABP5–Sp1 regulatory axis in sustaining AR signaling and enzalutamide resistance. Analysis of TCGA-PRAD datasets demonstrated that FABP5 expression is significantly elevated in prostate adenocarcinoma compared with normal prostate tissue (Fig. 1a; P < 0.001) and increases progressively with higher Gleason score, with significant upregulation observed across Gleason score 6 (P < 0.01), Gleason score 7 (P < 0.0001), Gleason score 8 (P < 0.01), and Gleason score 9 (P < 0.001) (Fig. 2a), supporting its strong association with aggressive disease and tumor progression. These observations are consistent with previous clinical studies reporting associations between elevated FABP5 expression and adverse clinical outcomes, including disease progression, metastasis, and poor prognosis in PC patients. In contrast, AR expression showed a modest but significant reduction in tumor samples compared with normal tissue (Fig. 1b; P < 0.01), while its variation across Gleason score groups was limited, with only Gleason score 6 reaching statistical significance (P < 0.01) (Fig. 2b), suggesting a more context-dependent role in disease progression. Similarly, Sp1 expression was moderately decreased in tumor samples relative to normal tissue (Fig. 1c; P < 0.01) and displayed variable expression across Gleason score categories, with significant differences observed in Gleason score 6 (P < 0.001), Gleason score 7 (P < 0.05), and Gleason score 9 (P < 0.001), but without a consistent progressive trend (Fig. 2c). Collectively, these findings highlight FABP5 as the most consistently and significantly dysregulated component of this axis at the clinical level, while AR and Sp1 exhibit more heterogeneous and context-dependent expression patterns across disease states.

Importantly, pharmacological inhibition of Sp1 significantly impaired aggressive tumorigenic phenotypes in 22RV1 cells. Mithramycin A treatment markedly reduced anchorage-independent colony formation in soft agar (Fig. 3a, b; ****P < 0.0001) and substantially decreased cellular invasive capacity (Fig. 3c, d; ***P < 0.001), indicating that disruption of the FABP5–Sp1 axis suppresses functional characteristics associated with malignant progression. These findings further support a broader role for the FABP5–Sp1 pathway in sustaining aggressive CRPC behavior beyond AR signaling alone.

Our in vitro findings demonstrate that FABP5 plays a critical role in stabilizing AR signaling under therapeutic pressure. Genetic ablation of FABP5 resulted in a marked reduction of AR-V7 expression in 22Rv1 cells (Fig. 4a, b) and significantly enhanced cellular responsiveness to enzalutamide, leading to near-complete suppression of AR-V7 and substantial reduction of full-length AR (Fig. 4a, b). These data provide direct evidence that FABP5 contributes to the persistence of AR signaling and resistance to AR-targeted therapy in CRPC cells, extending previous reports linking FABP5 to PC progression and poor prognosis. Notably, FABP5 ablation markedly enhanced sensitivity to enzalutamide, leading to near-complete suppression of AR-V7 and substantial reduction of full-length AR. This finding highlights a previously underappreciated role of FABP5 in maintaining therapeutic resistance and suggests that targeting FABP5 may restore responsiveness to AR-directed therapies in CRPC.

Importantly, our data identify Sp1 as a key transcriptional node connecting FABP5 to AR signaling. Gain- and loss-of-function analyses revealed that FABP5 positively regulates Sp1 protein expression: FABP5 overexpression significantly increased Sp1 levels (Fig. 5a, b), whereas FABP5-KO resulted in a pronounced reduction of Sp1 expression (Fig. 5c, d). Interestingly, although TCGA-PRAD analysis did not demonstrate a consistent positive association between FABP5 and Sp1 mRNA expression, our experimental findings revealed clear modulation of Sp1 protein levels by FABP5. This discrepancy may reflect post-transcriptional or post-translational regulatory mechanisms, as mRNA abundance does not necessarily correlate with protein expression. Previous studies have reported that Sp1 activity and stability can be regulated through multiple mechanisms, including phosphorylation, protein–protein interactions, and altered protein turnover. Therefore, the present findings suggest that FABP5 may influence Sp1 predominantly at the protein level, although the precise mechanism warrants further investigation. These findings support a regulatory relationship between FABP5 and Sp1 and suggest that FABP5 may modulate AR signaling indirectly through Sp1-dependent transcriptional mechanisms. While our data demonstrate strong regulatory associations between FABP5, Sp1, and AR signaling, further studies using ChIP-qPCR and luciferase reporter assays are required to confirm direct transcriptional binding and regulatory mechanisms at the promoter level. Consistent with this model, pharmacological inhibition of Sp1 using mithramycin A produced coordinated suppression of the FABP5–AR signaling axis. Mithramycin A treatment significantly reduced both AR and AR-V7 protein levels (Fig. 6a, b) and almost completely abolished FABP5 expression (Fig. 6c, d), demonstrating that Sp1 is indispensable for maintaining this transcriptional circuit in CRPC cells. These findings position Sp1 as a central regulator integrating metabolic and transcriptional signals that sustain AR activity under androgen-deprived conditions. Although mithramycin A is widely used as an Sp1 inhibitor, it may exert broader transcriptional effects beyond Sp1 suppression. Therefore, some of the observed molecular and phenotypic changes may also reflect additional transcriptional mechanisms, which should be considered when interpreting these findings. Beyond AR signaling, Sp1 inhibition also disrupted downstream lipid-regulated oncogenic pathways (Fig. 7a–d). Mithramycin A significantly reduced total PPARγ expression (Fig. 7a, b) as well as phosphorylated PPARγ levels (Fig. 7c, d), linking Sp1 activity to PPARγ-dependent lipid signaling in CRPC cells. In addition, the observed reduction in VEGFA expression following mithramycin A treatment (Fig. 7e, f; **P < 0.01) further links this axis to angiogenic signaling, suggesting that the FABP5–Sp1 pathway contributes not only to transcriptional and metabolic regulation but also to tumor-associated angiogenesis. Given the established role of PPARγ in lipid metabolism and PC progression, these data suggest that the FABP5–Sp1 axis coordinates both transcriptional and metabolic programs that support tumor survival and therapeutic resistance. Collectively, our findings support a model in which FABP5, through Sp1, stabilizes a transcriptional–metabolic signaling circuit that sustains AR and AR-V7 activity and promotes resistance to enzalutamide (Figs. 4–7). Disruption of either FABP5 or Sp1 was sufficient to destabilize this circuit, highlighting the therapeutic potential of targeting this pathway.

### Conclusion

This study identifies the FABP5–Sp1 regulatory axis as a critical determinant of sustained AR and AR-V7 signaling in CRPC. By integrating clinical transcriptomic analyses with mechanistic validation, we demonstrate that FABP5 is associated with aggressive disease features and contributes to enzalutamide resistance through Sp1-dependent regulation of AR signaling. Pharmacological inhibition of Sp1 disrupts this axis, leading to suppression of AR, AR-V7, FABP5, and downstream PPARγ-mediated lipid signaling. Collectively, these findings establish a mechanistic link between lipid metabolism, transcriptional regulation, and AR signaling in CRPC, and suggest that the FABP5–Sp1 axis may serve as both a potential therapeutic target and a clinically relevant biomarker. Targeting this pathway, alone or in combination with existing AR-directed therapies, may provide a promising strategy to overcome treatment resistance and improve outcomes in patients with advanced PC.

## Data Availability

The authors declare that data supporting the findings of this study are available within the article.

## Abbreviations

ADT: androgen deprivation therapy
AR: androgen receptor
AR-V7: androgen receptor splice variant 7
DMSO: dimethyl sulfoxide
FABP5: fatty acid binding protein 5
FABP5-KO: FABP5 knockout
FBS: fetal bovine serum
PPARγ: peroxisome proliferator-activated receptor gamma
p-PPARγ: phosphorylated PPARγ
PRAD: prostate adenocarcinoma
Sp1: specificity protein 1
VEGFA: vascular endothelial growth factor A
